# Statistical Optimization of Process Parameters for Lipase-Catalyzed Synthesis of Triethanolamine-Based Esterquats Using Response Surface Methodology in 2-Liter Bioreactor

**DOI:** 10.1155/2013/962083

**Published:** 2013-11-10

**Authors:** Hamid Reza Fard Masoumi, Mahiran Basri, Anuar Kassim, Dzulkefly Kuang Abdullah, Yadollah Abdollahi, Siti Salwa Abd Gani, Malahat Rezaee

**Affiliations:** ^1^Department of Chemistry, Faculty of Science, Universiti Putra Malaysia, 43400 Serdang, Selangor, Malaysia; ^2^Material Synthesis and Characterization Laboratory, Institute of Advanced Technology, Universiti Putra Malaysia, 43400 Serdang, Selangor, Malaysia; ^3^Institute of Bioscience, Universiti Putra Malaysia, 43400 Serdang, Selangor, Malaysia

## Abstract

Lipase-catalyzed production of triethanolamine-based esterquat by esterification of oleic acid (OA) with triethanolamine (TEA) in *n*-hexane was performed in 2 L stirred-tank reactor. A set of experiments was designed by central composite design to process modeling and statistically evaluate the findings. Five independent process variables, including enzyme amount, reaction time, reaction temperature, substrates molar ratio of OA to TEA, and agitation speed, were studied under the given conditions designed by Design Expert software. Experimental data were examined for normality test before data processing stage and skewness and kurtosis indices were determined. The mathematical model developed was found to be adequate and statistically accurate to predict the optimum conversion of product. Response surface methodology with central composite design gave the best performance in this study, and the methodology as a whole has been proven to be adequate for the design and optimization of the enzymatic process.

## 1. Introduction

Triethanolamine- (TEA-) based esterquat has been the primary ingredient in European fabric softeners and is becoming the global molecule of choice for various industries [1]. Esterquat cationic surfactant was considered as a type of biodegradable material utilized as a textile softening agent. There have been an increasing number of researchers who concern the biodegradable esterquat cationic surfactant since the beginning of the 1990s [2]. In addition, they are highly biodegradable and biocompatible because their ester bonds are easily hydrolyzed [3–5]. Besides biodegradability additional advantages such as excellent softening properties, suitability for various fabrics, and simple preparation procedures have been discovered by the use of esterquat cationic surfactants as textile softening agents [2].

In this work, triethanolamine and oleic acid were chosen as substrates to design an optimal model reaction which will lead to high conversion rate utilizing lipase from *Candida antarctica* (Novozym 435) as a biocatalyst in the organic solvent system. The investigated reaction conditions included enzyme amount, reaction time, reaction temperature, the molar ratio of substrates, and agitation speed. The major aim of this study was to model the effect of process parameters on the reaction yield. The most important stages in a process were modeling and optimization to improve a system and increase the efficiency of the process without increasing the cost. All process parameters are selected to conduct the optimization by using response surface methodology (RSM) [6–10]. The optimization of process has been reported by artificial neural network (ANN) in our previous study [11]. Subsequently, the simulated result in optimum conditions from the response surface methodology (RSM) and ANN was compared.

Prior to doing the statistical analyses by RSM, experimental data were inspected and explored the nature of variables by several normality tests. It has been observed that only a few researchers have paid attention to the application of right and accurate statistical techniques in order to validate experimental data [12]. Statistical methods are based on various underlying assumptions. One common assumption is that a random variable is normally distributed. In many statistical analyses, normality is often conveniently assumed without any empirical evidence or test. However, normality is critical in many statistical methods. Testing of assumptions usually involves obtaining descriptive statistics on variables [13]. Descriptive statistics provide important information about variables to be analyzed. Mean, median, and mode measure central tendency of a variable. Measures of dispersion include variance, standard deviation, and range. Researchers may draw a histogram, stem-and-leaf plot, or box plot to see how a variable is distributed [14]. When this assumption is violated, interpretation and inference may not be reliable or valid [15]. The usual processes in the statistical assessment of a data set are as follows: screen the data for outliers or blunders, plot the data to detect asymmetry and tail weight, calculate the indices of sample shape (i.e., skewness and kurtosis), perform tests of normality, and if the data is normal use parametric statistics for further analysis [15]. In order to test the validity of a normal distribution, quantitative tests need to be employed, such as Kolmogorov-Smirnov, Liliefors, and Shapiro-Wilks. In this study, normality tests also included the Kolmogorov-Smirnov (Lilliefors modification) and the Shapiro-Wilk for checking the normal distribution validity of variables.

## 2. Methods

### 2.1. Materials

Novozym 435, *Candida antarctica* lipase B immobilized on a macroporous acrylic resin (10,000 propyl laurate units per gram), was purchased from Novo Nordisk A/S (Bagsværd, Denmark). The enzyme is a granular product with a particle size of 0.2–0.6 mm. The bulk density of Novozym 435 is 350–450 kg/m^3^. *n*-Hexane obtained from J. T. Baker (USA) was used as the organic solvent. Oleic acid and triethanolamine were purchased from Merck, Germany. All other chemicals used in this study were of analytical reagent grade.

### 2.2. Experimental Design

The optimization study was carried out in accordance with the experimental design with 5 factors and 5 levels with 50 experimental points. The fractional factorial designs consisted of 32 factorial points, 10 axial points (two axial points on the axis of each design variable at a distance of 1.75 from the design center) and eight center points. The generalized response surface model is shown by (1), and the variables and their levels selected for the study were represented in Table 1:

(1) $$\begin{array}{c} \left[Y=\;\beta_{0}+\overset{5}{\underset{i=1}{\sum }}\beta_{i}x_{i}+\overset{5}{\underset{i=1}{\sum }}\beta_{ii}x_{i}^{2}+\overset{4}{\underset{i=1}{\sum }}\overset{5}{\underset{j=i+1}{\sum }}\beta_{ij}x_{i}x_{j}+\varepsilon \right], \end{array}$$

where *Y* (conversion %) represents the response variable, *β*_0_ is the constant term, *β*_*i*_ represents the coefficients of the linear parameters, *x*_*i*_ represents the variables, *β*_*ii*_ represents the coefficients of the quadratic parameter, *β*_*ij*_ represents the coefficients of the interaction parameters, and *ε* is the residual associated to the experiments.

### 2.3. Enzymatic Esterification and Analysis of Samples

The reactions were performed in 2000 mL reactor, and specified volumes of hexane were added as solvent. The reactor consisted of a screw cap and a glass flask with a capacity of 2 liters and an inner diameter of 10 cm. A four-bladed impeller (4.5 cm in diameter) was immersed in the reaction mixture a 2 cm height from the bottom of the flask to provide agitation effect. The impeller was connected by a shaft to motor for speed controlling purpose. A baffle was connected to the cap and immersed in the reaction mixture. The reaction temperature was controlled by immersing reactor in a temperature-controlled water bath. The reactions were catalyzed by various amounts of Novozym 435 from 1.5 to 8.5% w/w of oleic acid for experimental design at different temperature (51.25–68.75°C) and agitation speed (137.5–662.5 r.p.m.) values. The studied ranges of the substrates were 708 mmol for OA as a constant amount, while concentrations of TEA were varied according to Table 1 for the experimental design. All experiments were carried out in the range of 2–30 h, as shown in Table 1. The basic points for the design were selected from a preliminary study in laboratory scale [16] by using Taguchi design (data not shown).

At the end of the reaction periods, 30 mL aliquot was withdrawn from the system using a syringe. The reaction sample was terminated by dilution with 10 mL of ethanol-acetone (50 : 50, v/v). The enzyme particles were then separated by filtration, and the remaining free acid in the reaction mixture was determined by titration of the aliquots of reaction mixture against standard NaOH. The amount of reacted acid was determined from the values obtained for the control (without enzyme) and test samples. The ester formed was expressed as equivalent to conversion of the acid [17]. The ester formation was confirmed by thin-layer chromatography (TLC) using chloroform : methanol (95 : 5) solvent system. Further identification for ester formation was carried out by FTIR (Perkin Elmer, model 1650) and gas chromatography/mass spectroscopy GC-MS on a Shimadzu (model GC 17A; model MS QP5050A, Tokyo, Japan) instruments. For purification of the product, after termination of the reaction, the enzyme was filtered and the solvent removed by evaporator under reduced pressure. Product in the remaining mixture was separated via silica gel (Kieselgel 60, Merck, particle size 0.063–0.200 mm) column chromatography (15 cm% 20 mm) using a dichloromethane/methanol (90/10, v/v) mixture as eluent. A sample made up of 1 : 1 (w/w) ratio of silica gel and the free solvent reaction mixture was deposited at the top of the column previously equilibrated with dichloromethane/methanol (90/10, v/v) mixture. Five milliliter fractions were collected and tested using thin layer chromatography to identify the product-rich portions. Such fractions were pooled and the solvent evaporated by a rotary evaporator. The purity of product was then checked with TLC and GC before FTIR analysis.

## 3. Results and Discussion

### 3.1. Testing Experimental Data for Normality

Normal is used to describe a symmetrical, bell-shaped curve, which has the greatest frequency of scores in the middle, with smaller frequencies towards the extremes [18]. Normality can be assessed to some extent by obtaining skewness and kurtosis values.

Table 2 shows descriptive statistics to check the skewness and kurtosis values for five variables at three levels of each of them. For other levels conversions percentage was constant when variables were placed in ±1.75 levels, and they have been omitted.

The results showed that skewness ranged between −0.925 and 0.532 (acceptable range of normality is between −2.0 and +2.0). The values of kurtosis ranged between −0.848 and 1.111 (acceptable range of normality is between −5.0 and +5.0) [19]. As a result, the skewness and kurtosis values indicate almost normal distribution. However, these descriptive statistics do not provide conclusive information about normality, and testing normality needs to use some other statistics tests. SPSS software provides two different statistics for testing normality. The Shapiro-Wilk and Kolmogorov-Smirnov tests were used for data distribution analysis. Both tests similarly demonstrated that the data set was normally distributed. As shown in Table 3, the *P* values of Shapiro-Wilk and Kolmogorov-Smirnov tests confirm null hypothesis that the variable are normally distributed (*P* ≥ 0.05). Since the number of observations is less than 2,000, however, Shapiro-Wilk test will be appropriate to this case. 

### 3.2. Data Processing and Analysis of Variance (ANOVA)

The results at each point based on experimental design for the enzymatic reaction of TEA-based esterquat are presented in Table 4. Evaluation of coefficients of the empirical models and their statistical analyses were carried out using central composite design.

**Figure 1:**
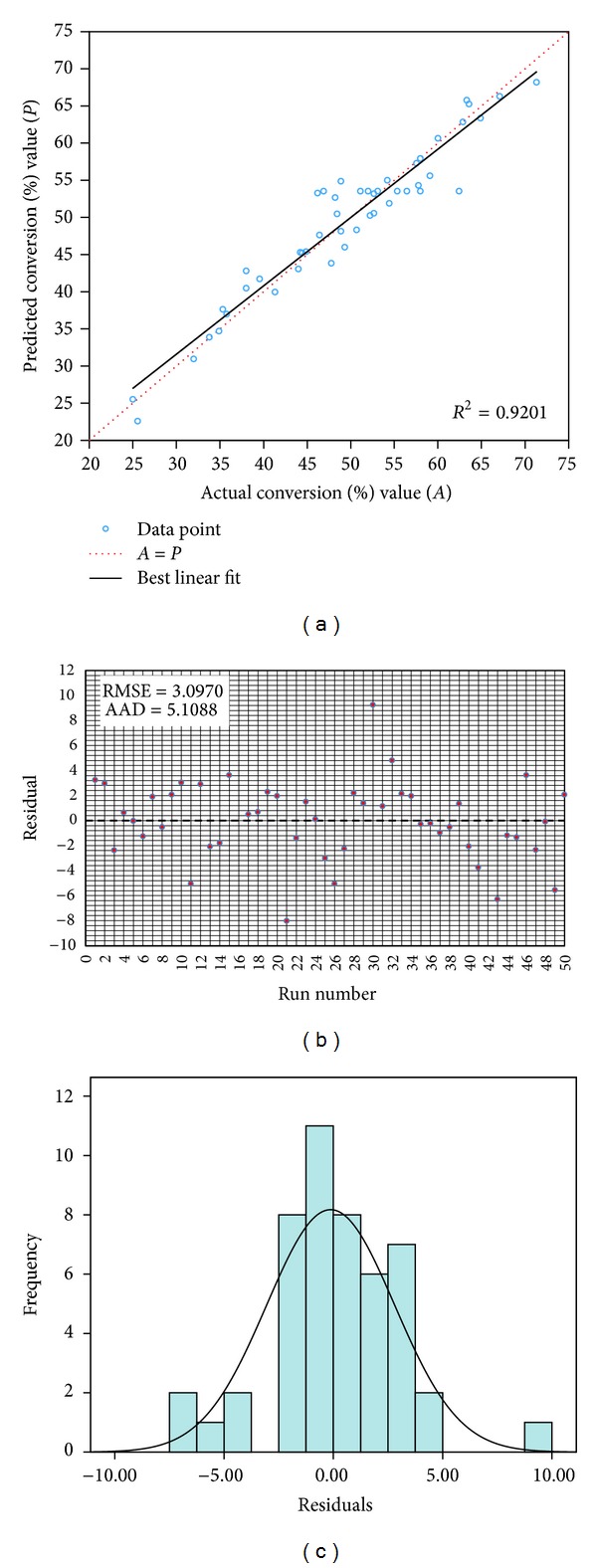
(a) Scatter plot of predicted conversion% value versus actual conversion% value (b) residual plot of runs from central composite design (c) histogram of residuals with normal overlay.

Fitting of the data to various models (linear, 2FI, quadratic, and cubic) and their subsequent analysis of variance showed that TEA-based esterquat synthesis was most suitably described with a quadratic model. The model was modified based on the insignificancy of some model terms. The final reduced model to predict the conversion % of TEA-based esterquat catalyzed by Novozym 435 is shown as follows:

(2) Y(Conversion%)=53.56+1.47X1 +5.76X2+6.75X3−6.13X4 +2.69X5+1.47X2X3+1.77X2X4 +1.93X3X4+1.49X4X5−1.85X12 −0.97X22−1.63X52,

where *Y* matches product conversion % and *X*_1_, *X*_2_, *X*_3_, *X*_4_, and *X*_5_ match to coded values for the enzyme amount (% w/w), reaction time (h), reaction temperature (°C), the molar ratio of substrates (mole), and agitation speed (r.p.m.), respectively. The positive sign in front of the terms indicates a synergistic effect while the negative sign indicates an antagonistic effect. Negative values of coefficient estimates denote negative influence of parameters on the reaction. It was observed that all the linear coefficients from the model gave positive effect except the coefficient estimate for the molar ratio of substrates (*X*_4_) in the model of percentage conversion. This may be due to that the percentage of conversion was negatively affected by the presence of the higher ratio of oleic acid as the ratio of oleic acid/triethanolamine. From the equation, the conversion of enzymatic reaction has linear and quadratic effects by the five process variables. The model was found to have coefficient of determination value (*R* ^2^) of 0.9201, which means that 92.01% of the total variation in the results was attributed to the independent variables investigated. When *R* ^2^ approaches unity, the better empirical model fits the actual data [20]. Normally, a regression model having an *R* ^2^ value higher than 0.9 was considered as model having a very high correlation [21]. Hence, the *R* ^2^ value in this regression model is relatively high, which indicates a good agreement between predicted and experimental conversion of TEA-based esterquat reaction.  Figure 1 summarizes correlation between experimental values and predicted values by using the developed model.

Figure 1(a) shows the actual values versus predicted values of the product conversion %, which indicated a good agreement between actual and predicted responses. A residual plot allowed visual assessment of the distance of each observation from the fitted line (Figure 1(b)). The residuals randomly scattered in a constant width band about the zero line.  Figure 1(c) shows the histogram of the residuals in allowed visual assessment of the assumption. As observed, the measurement errors in the response variable were normally distributed, and the histogram of the residuals revealed a normal distribution overlay.

Statistical analysis based on ANOVA for the response surface quadratic model is presented in Table 5. The *P* value for the model is less than 0.05, which indicates that it is a significant and desirable model. Besides, the value of *P* < 0.0001 indicates that there is only a 0.01% chance that a “model *F*-value” this large could occur due to noise in the experiments. The “Lack of Fit *F*-value” of 0.43 implies that lack of fit is not significant relative to pure error. Thus, it is possible quantitatively judge if the model represents the observations satisfactorily.

### 3.3. Mutual Effect of Process Parameters

**Figure 2:**
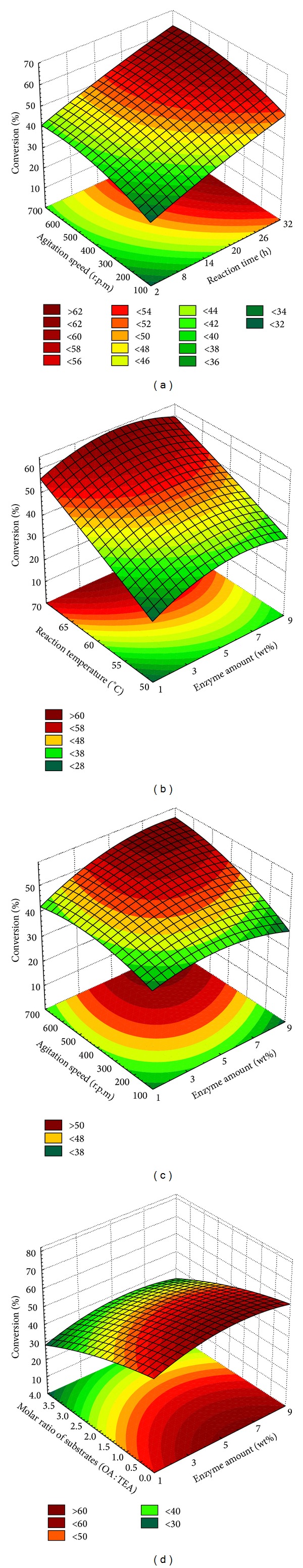
Response surface plots: (a) reaction time (h) versus agitation speed (r.p.m.); (b) enzyme amount (% w/w) versus reaction temperature (°C); (c) enzyme amount (% w/w) versus agitation speed (r.p.m.); (d) enzyme amount (% w/w) versus molar ratio of substrates (OA : TEA) (mole) on percentage conversion as response.

The terms in (2) show that interactions between variables have significant effect on the conversion% of enzymatic reaction of TEA-based esterquat. Therefore, instead of studying single variable the interactions will be investigated, which is significant and important for a comprehensive optimization study.  Figure 2(a) shows the effects of different reaction time and agitation speed on the conversion % of product in three-dimensional surface response. Generally, increased reaction time and agitation speed resulted in an increase percentage of conversion until agitation speed reached 523 r.p.m. The response started to decrease after the agitation speed exceeded 523 r.p.m. even at the higher reaction time. However, it was observed that reaction time showed a significant effect to the reaction conversion at the higher agitation speed. Increasing agitation speed had increased the external mass transfer rates between the bulk phase of the reaction mixture and surface of enzyme; moreover, higher reaction time also promoted collision time between enzyme and substrate molecules. As shown in Figure 2(b), the reaction with the enzyme amount of 5.80% w/w led to the maximum percentage of conversion. Response surface plot for interaction between enzyme amount and reaction temperature was generated with reaction time fixed at 16 h, the molar ratio of substrates (OA : TEA) 2 : 1 mole, and agitation speed 400 r.p.m. The percentage conversion of product increased by increase ongoing from 3 to 5.80% w/w and thereafter decreased with further increase to 7% w/w. However, higher temperatures tended to induce enzyme inactivation due to denaturation processes [22, 23]. These results were similar to those in most reviewed papers, namely, that Novozym 435 was optimally used at temperatures between 40°C and 60°C [24, 25].  Figure 2(c) represents the effect of varying amount of enzyme and agitation speed on the synthesis of TEA-based esterquat with constant condition for other independent variables (reaction temperature of 60°C, reaction time of 16 h, and substrate molar ratio of 2 : 1 mole). From Figure 2(c), while the enzyme amount and agitation speed increased, the conversion of esterquat was increased as the agitation speed reached 523 r.p.m. in the enzyme amount of 5.80% w/w. However, the effect of enzyme amount variable was lower than the effect of agitation speed variable. Increase in agitation speed caused the substantial increase in the specific interfacial area between the substrate and the enzyme present in the nonaqueous phase by reducing the droplet size [26, 27]. A negative effect in percentage of conversion was detected with agitation speed greater than 523 r.p.m. This may be due to adverse shear effect caused by impeller at higher agitation speed. Typically, the immobilized enzyme was driven radially from impeller against the wall of the reactor, forcing the breakage, especially at high agitation speed [28]. Finally, Figure 2(d) shows the effect of varying the amounts of enzyme and molar ratio of substrates on the esterification reaction of oleic acid and triethanolamine while reaction time and reaction temperature are fixed at 16 h and 60°C, respectively. It was shown that the maximum conversion of esterquat was obtained when the enzyme amount was 11.6 g and increased with the lower molar ratio of substrates. However, increase in acyl donor showed less significant increase in the esterification conversion, on the other hand, and resulted in slight decrease of percentage conversion at the high amount of enzyme 14 g and the molar ratio of substrates of 3 : 1 mole. This was due to the limiting factor caused by triethanolamine, which was significant at the high amount of oleic acid and hence reduced the percentage of conversion.

### 3.4. Optimization by Response Surface Methodology and Model Validation

The next step in the present study was to determine the effects of five independent variables (enzyme amount, reaction time, reaction temperature, molar ratio of substrates, and agitation speed) shown in Table 6, along with the mean predicted values for enzymatic reaction product. For this purpose, the response surface methodology, using a central composite design, was adopted for finding optimal conditions. Experiment was then carried out under the recommended conditions and resulting response was compared to the predicted values. The optimum reaction parameters were enzyme amount of 4.77% w/w, reaction time of 24 h, reaction temperature of 61.9°C, substrates molar ratio (OA : TEA) of 1 : 1 mole (0.708 mole of OA and TEA), and agitation speed of 480 r.p.m. Comparison between RSM and ANN methods was then assessed in optimum conditions point for enzymatic synthesis of TEA-based esterquat at 2000 mL scale. The reaction of experiment gave the reasonable percentage of conversion 63.57%. This result confirmed the validity of the model, and the experimental value was determined to be quite close to the predicted value (65.08%) in comparison with ANN result (61.14%), implying that empirical model derived from RSM experimental design can be used to adequately describe the relationship between the independent variables and response.

## 4. Conclusion

In the present paper, RSM was used to optimize the enzymatic reaction conditions. A central composite design was applied to optimize the experimental conditions for synthesis of TEA-based esterquat at 2000 mL scale. The normality test was investigated as an initial step in process capability studies for better results and higher accuracy. Considering normality tests, the results indicated that all of the data and distributions were close to expected values under normality. The variables include enzyme amount, reaction time, reaction temperature, substrates molar ratio, and agitation speed. Quadratic mathematical model was suggested for synthesis of TEA-based esterquat. Analysis of variance corroborates the accuracy of the model by using high *F* value (33.60), very low *P* value (<0.0001), nonsignificant lack of fit, and the coefficient of determination (*R* ^2^ = 0.9201). A conversion percentage of 63.57% was attained, which was good compared to the predicted amount of 65.08%, with the relative standard error percentage (RSE) 2.32%. The comparison of RSM and ANN (QP) indicated that the RSM had less RSE% rather than ANN (QP) method (3.98%). The methodology as a whole has proven that RSM is adequate for the design and optimization of the enzymatic process.

## Figures and Tables

**Table 1:** Variables and their levels employed in the central composite design.

Variables	Units	Coded level of variables				
		−1.75	−1	0	1	1.75
*X*_1_: Enzyme amount	w/w%	1.5	3	5	7	8.5
*X*_2_: Reaction time	h	2	8	16	24	30
*X*_3_: Reaction temperature	°C	51.25	55	60	65	68.75
*X*_4_: Molar ratio of substrates	OA : TEA (mole : mole)	0.25 : 1	1 : 1	2 : 1	3 : 1	3.75 : 1
*X*_5_: Agitation speed	r.p.m.	137.5	250	400	550	662.5

**Table 2:** Descriptive statistics to check the skewness and kurtosis values for five variables.

	Statistic for variables														
	Enzyme amount (w/w%)			Reaction time (h)			Reaction temperature (°C)			Molar ratio of substrates (mole)			Agitation speed (r.p.m.)		
	−1	0	1	−1	0	1	−1	0	1	−1	0	1	−1	0	1
Skewness	−0.261	−0.819	−0.524	−0.551	−0.608	−0.550	−0.446	−0.925	−0.540	0.532	−0.825	0.349	−0.425	−0.781	−0.220
Kurtosis	−0.706	−0.034	−0.163	−0.706	−0.766	−0.399	−0.848	0.671	−0.255	−0.263	1.111	−0.728	−0.737	−0.156	−0.476

**Table 3:** The Shapiro-Wilk and Kolmogorov-Smirnov tests for five variables.

Conversion (%)	Level	Kolmogorov-Smirnov			Shapiro-Wilk		
		Statistic	Df	*P* value	Statistic	Df	*P* value
Enzyme amount (% w/w)	3.00	0.120	16	0.200	0.962	16	0.694
	5.00	0.161	14	0.200	0.926	14	0.271
	7.00	0.188	16	0.133	0.967	16	0.784

Reaction time (h)	8.00	0.154	16	0.200	0.938	16	0.327
	16.00	0.122	14	0.200	0.975	14	0.930
	24.00	0.141	16	0.200	0.948	16	0.455

Reaction temperature (°C)	55.00	0.174	16	0.200	0.936	16	0.308
	60.00	0.157	15	0.200	0.920	15	0.194
	65.00	0.133	16	0.200	0.966	16	0.769

Molar ratio (OA : TEA) (mole)	1.00	0.137	16	0.200	0.957	16	0.609
	2.00	0.121	15	0.200	0.958	15	0.651
	3.00	0.158	16	0.200	0.960	16	0.666

Agitation speed (r.p.m.)	250.00	0.155	16	0.200	0.946	16	0.431
	400.00	0.146	14	0.200	0.932	14	0.330
	550.00	0.104	16	0.200	0.971	16	0.856

**Table 4:** Central composite design matrix (coded) and result for the model of TEA-based esterquat synthesis.

Run no.	Enzyme amount (w/w%)	Reaction time (hour)	Reaction temperature (°C)	Molar ratio of substrates (mole)	Agitation speed (r.p.m.)	Conversion %	
						Actual	Predicted
1	0	0	0	0	0	56.44	53.56
2	0	0	0	0	−1.75	47.78	43.86
3	−1	1	−1	−1	1	48.22	52.69
4	−1	−1	−1	−1	1	46.44	47.63
5	1	−1	1	−1	1	57.56	57.28
6	−1	1	1	−1	1	63.56	65.25
7	1	−1	−1	−1	−1	48.89	48.17
8	−1	1	1	−1	−1	62.89	62.84
9	1	−1	1	1	1	50.67	48.33
10	−1	1	−1	1	1	44.00	43.07
11	0	0	−1.75	0	0	39.56	41.75
12	−1	−1	−1	1	1	32.00	30.96
13	0	0	0	0	0	51.11	53.56
14	−1	−1	−1	−1	−1	44.44	45.23
15	−1	−1	1	−1	1	57.78	54.33
17	0	1.75	0	0	0	60.00	60.65
18	1	−1	−1	1	−1	25.00	25.53
19	1	−1	−1	−1	1	52.67	50.58
20	−1	−1	−1	1	−1	25.56	22.59
21	0	0	0	0	1.75	46.22	53.29
22	1	1	1	−1	−1	63.33	65.78
23	−1	1	−1	−1	−1	52.22	50.27
24	1	−1	−1	1	1	33.78	33.90
25	1.75	0	0	0	0	48.44	50.48
26	0	0	0	1.75	0	38.00	42.84
27	1	1	−1	1	−1	35.33	37.64
28	1	1	−1	−1	1	59.11	55.62
29	−1	−1	1	−1	−1	54.44	51.92
30	0	0	0	0	0	62.44	53.56
31	1	1	1	1	−1	58.00	57.94
32	0	0	0	0	0	58.00	53.56
33	0	0	0	0	0	55.33	53.56
34	−1	1	1	1	1	64.89	63.37
35	−1	−1	1	1	1	44.89	45.38
36	−1	1	−1	1	−1	34.89	34.70
37	1	1	1	1	1	67.11	66.32
38	1	1	−1	−1	−1	52.67	53.22
39	1	1	1	−1	1	71.33	68.19
40	−1.75	0	0	0	0	44.22	45.32
41	1	−1	1	−1	−1	48.89	54.87
43	0	0	0	0	0	46.89	53.56
44	0	0	0	0	0	52.00	53.56
45	−1	1	1	1	−1	54.22	55.00
46	1	−1	1	1	−1	41.33	39.96
47	−1	−1	1	1	−1	35.78	37.02
48	0	0	0	0	0	53.11	53.56
49	0	−1.75	0	0	0	38.00	40.50
50	1	1	−1	1	1	49.33	46.01

**Table 5:** ANOVA for the quadratic model developed for synthesis of TEA-based esterquat.

Source	Sum of squares	DF	Mean square	*F* value	*P* value
Model	4892.48	12	407.71	33.60	<0.0001
Residual	424.71	35	12.13	—	—
Lack of fit	267.43	28	9.55	0.43	0.9494
Pure error	157.27	7	22.47	—	—
Corr. total	5317.19	47	—	—	—

**Table 6:** Optimum conditions derived by RSM for synthesis of TEA-based esterquat.

Methods	Optimal conditions					Conversion %		
	Enzyme amount (w/w%)	Reaction time (h)	Reaction temperature (°C)	Molar ratio of substrates (mole)	Agitation speed (r.p.m.)	Actual	Predicted	RSE%
RSM	4.77	24	61.9	1 : 1	480	63.57	65.08	2.32
ANN (QP)	4.77	24	61.9	1 : 1	480	63.57	61.14	3.98
